# Improving metabolic risk in patients with mental illness through ‘mental health care plans’ in primary health care

**DOI:** 10.1177/00048674251337030

**Published:** 2025-05-13

**Authors:** Mithilesh Dronavalli, Andrew Page, Shahana Ferdousi, Max Osaghae, Sandro Sperandei

**Affiliations:** 1 Translational Health Research Institute, Western Sydney University, Campbelltown, NSW, Australia; 2 Western Sydney Primary Health Network, Westmead, NSW, Australia

**Keywords:** Metabolic risk, mental illness, social disadvantage, care plans, primary care

## Abstract

**Aim:**

To determine the association between types of mental illness, levels of social disadvantage and metabolic risk factors (obesity, tobacco smoking, high blood pressure and high cholesterol) and to investigate whether mental health care plans modify metabolic risk.

**Methods:**

Two cohorts (2016–2023) of all primary care patients in Western Sydney with active mental illness or never having a mental illness (reference cohort) were compared on metabolic risk and change in metabolic risk during the period of the care plan (12 months) using random effects regression. Also, the social gradient of metabolic risk in patients with active mental illness was determined. Analyses were adjusted for age, sex and social disadvantage.

**Results:**

There were 29,592 patients with active mental illness and 962,416 never having mental illness. Care plan utilisation ranged from 35% to 51%, with the lowest utilisation for Schizophrenia (33%). Daily tobacco smoking rates were elevated for all mental illness types. Care plans were associated with a reduction in daily tobacco smoking rates (0.7 odds ratio; 95% confidence interval: 0.6–0.99). Patients with schizophrenia had excess body mass index (+5.6 body mass index; 95% confidence interval: 2.1–9.1). Care plans reduced the excess body mass index (−6.6 body mass index; 95% confidence interval: −17.7 to +4.5)). Obesity and daily tobacco smoking followed a social gradient in patients with mental illness, but cholesterol and blood pressure did not. High blood pressure and high cholesterol was not elevated compared to the reference group in all types of mental illness.

**Conclusion:**

Metabolic risk was particularly elevated in tobacco smoking rates for patients with any active mental illness and for obesity in patients with schizophrenia. Care plans were associated with a reduction in much of this risk.

## Introduction

Patients diagnosed with mental illness have challenges across many domains of life, including career, relationships, education, finance and more. However, there is also a risk to physical health through their circumstances, medication, and the mental illness itself (McGinty et al., 2016). Metabolic and cardiovascular risk factors highlight this risk to physical health. For example, patients taking types of psychotropic medication such as atypical anti-psychotics can develop obesity, have an increased risk of developing diabetes, have poor diabetic control and have increased cholesterol (McGinty et al., 2016). In addition, these risk factors–and mental illness prevalence–are differentially distributed by socio-economic disadvantage. For example, socio-economic disadvantage is associated with a higher prevalence of tobacco smoking and the use of illicit drugs, which can compound this metabolic risk (Barbeau et al., 2004), and mental illness can lead to declines in socio-economic status (Murphy and Daumit, 2023).

In Australia, the general practitioner (GP) oversees the care of patients with mental illness with appropriate referrals to psychiatry, psychology and other medical specialists and allied health care providers (Services Australia, 2024). GPs in Australia are provided funding to develop ‘care plans’ for patients with mental illness to proactively improve and maintain health and prevent the development of poor physical health outcomes (Victorian Department of Health, 2023). Care plan funding in Australia is substantial, with GPs funded $158.80 (AUD) per patient from the government for each consultation over the 12-month period of a care plan (Department of Health and Aged Care, 2024).

To date, there has been no previous research that evaluates the association between care plans in modifying metabolic risk factors. Previous literature shows that care plan formulation is different for different types of mental illness and also for physical illness (Banfield et al., 2019). Patients with depression and anxiety generally have a higher percentage of care plan formulation than those with bipolar disorder, schizophrenia or diabetes (Banfield et al., 2019). The association between care plans for metabolic risk has previously been assessed in an audit of 230 patients with type 2 diabetes, which showed that adherence to diabetes guidelines increased during the period of the care plan (Zwar et al., 2007).

Accordingly, this study investigates the association of care plans in reducing metabolic risk factors among all patients with mental illness attending a GP setting over a maximum 7-year follow-up period in the socio-economically diverse geographic catchment of Western Sydney. Specifically this study investigated (1) the association between selected mental illness (anxiety, bipolar disorder, depression, schizophrenia and attention deficit hyperactivity disorder (ADHD)) and key metabolic risk outcomes (obesity, tobacco smoking, high blood pressure, high cholesterol), (2) the association between care plan use and change in metabolic risk outcomes for each mental illness type and (3) the pattern of care plan use for selected mental illness and also the distribution of metabolic risk by socio-economic status and geographic area.

## Methods

### Study design and setting

In Australia, Primary Health Networks (PHNs) oversee and commission primary care programmes and general practice clinics for local areas. Western Sydney Primary Healthcare Network (WSPHN) covers the geographic catchment of Western Sydney. It comprises the Local Government Areas of Blacktown, Cumberland, Parramatta and the Hills Shire (a population of ~1M at 2021 census). This study employed a retrospective cohort study design comprising two cohorts: a cohort of all patients with a diagnosed active mental illness who attended a General Practitioner in WSPHN over the period 2 January 2016 to 27 January 2023 (*N* = 29,592 patients, representing 93,359 GP visits); and a cohort of all patients without a previously documented mental illness who also had not previously been prescribed mental health medication in the same period (N = 962,416 patients, representing 3,738,854 GP visits). Individuals were included if they were over the age of 18, as the metabolic profiles of children and adults are different from older age cohorts. Mental illness types included only ADHD, Anxiety, Bipolar, Depression and Schizophrenia. These mental illnesses were selected as they represent high prevalence disorders in the PHN or are outcomes that often require medication that can further affect metabolic risk (schizophrenia, bipolar disorder). These diagnoses were recorded as checked items by the GP. Those patients with inactive mental illness or those taking mental health medication without a diagnosis of mental illness (as listed previously) that were investigated were excluded from both cohorts. A complete case analysis was carried out due to the large number of patients and limited missing data.

Data were automatically gathered via clinical electronic medical record systems at the GP, which are streamlined and de-identified at the individual record level. This data is typically collected for reporting to the Australian Department of Health. This system is referred to as the Practice Aggregation Tool for the Clinical Audit Tool – Business Intelligence (PAT-BI) and the recording of metabolic indicators on a regular basis is known as Practice Incentives Programme – Quality Improvement (PIP-QI). They included the variables: De-identified patient ID, visit sequence, age, sex, Body Mass Index (BMI), total cholesterol, being a daily tobacco smoker, systolic blood pressure, type of Mental Illness, post-code of GP practice and associated Socio-economic Index for Areas (SEIFA) score. These data were accessed by ethics approval from the Western Sydney Local Health District Human Research Ethics Committee (Project ID: 2021/PID00633 Ethics Ref: 2021/ETH00550 Governance Ref: 2021/STE01014).

### Outcomes

The primary outcome was a change in metabolic risk over time as determined by a series of outcomes. These included and were limited to BMI, obesity (BMI ⩾ 30), total cholesterol, high cholesterol (defined as a total cholesterol > 6.2 mmol/L), being a daily tobacco smoker and having high blood pressure (systolic ⩾ 140). These risk factors were collected and coded in the PAT-BI as part of the metabolic screening of existing primary health care patients, as recommended by the Department of Health, with reminders installed in the GP medical record management software. GPs receive additional funding to collect data on the following metabolic outcomes: Metabolic screening included annual BMIs, 6-monthly HbA1c for patients with Diabetes and cholesterol levels (recorded annually for patients with normal cholesterol and 6-monthly for those with elevated cholesterol). The metabolic indicators are the only regularly collected indicators in the PIP-QI system.

### Exposure

There were two primary exposures in the current study: (1) those with an active mental illness vs those never having a mental illness and not taking psychotropic medication among the cohort of those with a mental illness and (2) those prescribed a care plan and those not prescribed a care plan. For (1), an active mental illness was defined as having a diagnosis of ADHD, Anxiety, Bipolar Disorder, Depression or Schizophrenia. Those with inactive mental illness was defined as the patient’s GP documenting that the patient did not have an active mental illness of the type previously listed in the clinical management software., the primary exposure was being prescribed a Medicare Benefits Schedule (MBS) Care Plan (MBS care plan item numbers: 2700 to 2799 (face to face) and 92,100 to 92,199 (via telehealth)). These item numbers from MBS are used to claim funding from the Federal Government.

Care plan formulation was summarised by counting all MBS item numbers listed above. Care Plans are valid for 12 months from the initial consultation where the care plan was prescribed. Data were coded by each GP visit, and all visits within 12 months of receiving a care plan were considered to be under the care plan period. If another care plan was carried out, this care plan period was extended by another 12 months from the most recent care plan. This way, all patients were categorised as either on a care plan or not on a care plan.

### Other study factors

Identified available confounders included age (18–38 years, 38–58 years, 58–78 years and above 78 years), sex and area socio-economic status. Socioeconomic status was measured by the SEIFA of the GP clinic’s postcode. SEIFA is measured at Statistical Area 1 (SA1), which is a smaller geographical area than a postcode. However, the Australian Bureau of Statistics (ABS) has stated that there are no significant mismatches between the SEIFA scores of postcodes in Western Sydney and their constituting SA1s (Australian Bureau of Statistics, 2023). SEIFA scores were categorised into population quintiles, representing areas from lowest to highest socio-economic status (SES).

### Analysis

Descriptive statistics were calculated for those under a care plan and compared to those not under a care plan by age, sex, SES quintile and type of active mental illness. Longitudinal analyses over a maximum patient follow-up period of 7 years investigated the association between being on a care plan and each metabolic outcome by (1) type of mental illness and (2) socio-economic status quintile. The period from establishing a care plan to 12 months after this index date was defined as the care plan period. If the care plan was renewed at the end of 12 months, this period was extended for another 12 months and considered the same care plan.

When stratifying by type of mental illness, only those with active symptoms on a care plan were classified as the exposed group. Those with inactive symptoms of that mental illness were excluded (Supplementary Table 1). Patients who attended GPs without mental illness and who had never been prescribed mental health medications were the referent group.

Differences in metabolic risk among mental illness types were carried out using separate logistic regression for each mental illness type. For each regression analysis, the outcome was any of the metabolic risk factors separately (obesity, tobacco smoking, high blood pressure, high cholesterol) and each mental illness type separately (ADHD, anxiety, bipolar disorder, depression and schizophrenia), adjusted for age in categories and SEIFA quintiles in categories, while accounting for correlated observations over time within the same patient using (‘xtset’ and ‘xtlogit’ in Stata). The model was structured so that the exposed group was those with the specific mental illness type, and those who were unexposed, came from the reference cohort of those never having a mental illness and no recorded use of psychotropic medication.

Ever smoking tobacco daily implies that the ‘Daily Tobacco Smoker’ flag was ticked at any GP visit in the reference period. Also when analysing smoking, this study investigated alternating states of either being a ‘Daily Tobacco Smoker’ or not being a ‘Daily Tobacco smoker’ and this was analysed longitudinally using xtlogit (longitudinal logistic regression) to take into account within patient tobacco smoking states over time. The reduction in tobacco smoking was investigated through an interaction between the specific mental illness and the care plan period indicator.

Care Plan use was calculated by counting the number of unique patients on a care plan and deriving a percentage for each mental illness type, the overall cohort of active mental illness and the reference cohort.

Differences in metabolic risk over time between those receiving a care plan and those not receiving a care plan were analysed using random effects generalised least squares regression (‘xtreg’ in Stata) for BMI as a continuous measure and random effects logistic regression giving an odds ratio (OR) (‘xtlogit’ in Stata) for binary metabolic risk factors to calculate a relative risk estimate. Separate models were specified for each type of metabolic outcome, stratified by mental illness type.

The association between social disadvantage as measured by SEIFA quintiles and each metabolic risk factor as the outcome was carried out for only patients with active mental illness. The commands ‘xtset’ and ‘xtlogit’ were used as they account for correlated outcomes by patient over time. No adjustments were made for SEIFA as it is the level of social disadvantage in the area of the patient rather than being an individual patient variable, which limits potential confounding. All modelling was based on a complete case analysis, as the high degree of missingness and limited number of available covariates (age, sex, SEIFA, mental illness type, metabolic outcome and care plan period) precluded the use of multiple imputation (Hughes et al., 2019). However, for the main socio-economic status variable (based on SEIFA code) there were no substantial systematic differences by exposures and outcomes (Supplementary Tables 2 and 3).

Choropleth maps were also constructed using ‘spmaps’ to show the overall metabolic risk and association with care plans based on the effect size from the above care plan model at a postcode level using ‘statsby’ in Stata.

## Results

Characteristics of the patients in this cohort for available socio-demographic variables (sex, age and area socio-economic status) and mental health care plan use are provided in Table 1.

**Table 1.** Care plan prescription among different patients with active mental illness aged above 18 years in Western Sydney by different types of mental illness, social disadvantage quintiles, age and sex.

Mental illness\ever on a care plan	Yes	%Yes	All patients	No mental illness and not taking MH medication	Yes	%Yes	All patients
Social disadvantage (SEIFA Quintile)	Yes	%Yes	All patients	Social disadvantage (SEIFA Quintile)	Yes	%Yes	All Patients
1 – Most disadvantaged	2418	43%	5650	1	164,293	19%	851,576
2 – Moderate to most disadvantage	1672	43%	3934	2	137,521	16%	851,576
3 – Moderate disadvantage	3847	51%	7565	3	157,701	19%	851,576
4 – Mild disadvantage	2159	45%	4760	4	140,675	17%	851,576
5 – Least disadvantaged	6321	54%	11,809	5	251,386	30%	851,576
Mean age in years (SD)	40 (SD: 15)		42 (SD: 16)	Mean age in years (SD)	47 (SD: 18)		
Median age (IQR)	37 (IQR: 28–49)		40 (IQR: 29–53)	Median age (IQR)	43 (IQR: 33–60)		
Males	9134	51%	18,015	Males	457,508	48%	958,370
Females	5846	51%	11,440	Females	500,592	52%	958,370
Non-binary	3	50%	6	Non-binary	270	0%	958,370

Data from 2016 till 2023.

Those with schizophrenia had a very high risk of obesity, with an average BMI score over 5 points higher than the reference cohort without mental illness (*β* = 5.6: 95%CI: 2.1;9.1) (Table 2). The prevalence of obesity was 45% among those with schizophrenia compared to 31% among those never having mental illness. The BMI in this reference cohort having no mental illness was 28.1. Similarly, those with bipolar disorder had an increased risk of obesity with a higher average BMI than the reference cohort (*β* = 2.0, 95% CI: −1.9; +6.0), and the prevalence of obesity was 36% among those patients with bipolar disorder and 31% in the reference cohort.

**Table 2.** Comparing metabolic outcomes between different mental illness diagnoses and those without mental illness, adjusted for age and social disadvantage from 2016 till 2023.

Metabolic risk factors	Total patients (n)	GP visits during follow-up	%n on a mental health care plan	Obesity		Difference in BMI^a^	Daily tobacco smoking		High BP		High cholesterol		Difference in total chol^a^
Mental illness type				Prev.	OR	β	Prev.	OR	Prev.	OR	Prev.	OR	β
Any mental illness	29592	91349	51%	27%	1.5 (0.9;2.6)	0.3 (−.3;0.8)	14%	2.4 (2.1:2.7)	15%	1.1(0.9;1.3)	11%	1.4(0.9;2.0)	0.01(−0.05; 0.06)
Anxiety	19894	57030	51%	25%	1.2 (0.7;2.2)	0 (−0.6;0.6)	13%	2.0 (1.7;2.4)	13%	1.2 (0.9;1.5)	11%	1.3 (0.8;2.1)	0.01 (−0.07;−0.08)
Bipolar	567	1798	40%	36%	^b^	2.0 (−1.9;6)	23%	6.5 (3.1;13.4)	14%	1.1 (0.3;3.8)	5%	0.2 (0;8.6)	–0.23 (−0.69;−0.24)
Depression	10049	30635	49%	29%	1.6 (0.7;3.7)	0.6 (−0.3;1.5)	16%	3.0 (2.5;3.7)	15%	1.0 (0.8;1.4)	12%	1.4 (0.7;2.6)	0.04 (−0.07;−0.14)
Schizophrenia	473	1369	33%	45%	^b^	5.6 (2.1;9.1)	24%	4.7 (2.3;9.9)	19%	0.9 (0.3;3.1)	7%	0.3 (0;−21.3)	–0.44 (−0.98;−0.1)
ADHD	668	1718	35%	33%	1.0 (0.04;25.6)	3.0 (−0.9;6.9)	13%	2.0 (0.9;4.2)	10%	0.8 (0.2;2.9)	11%	2.4 (0.1;−40.2)	0.35 (−0.13;−0.83)
No mental illness^c^	962416	3738854	NA	31%	1	25.4	9%	1	17%	1	10%	1	4.7

a Some patients may have more than one mental illness. Beta-coefficients for random effects linear regression model. ORs presented from random effects logistic regression models compare those with a care plan to those without one. ^b^Insufficient Variation in Data. Prev.: Period Prevalence over the follow-up of the metabolic risk factor among patients with active mental illness in Western Sydney. ^c^Referent group: Those without a previously documented mental illness who also had not previously been prescribed mental health medication.

The reference cohort had a 9% prevalence of ever smoking tobacco daily during the follow-up. For patients with bipolar disorder, this prevalence was 23%; for schizophrenia, it was 24%; and the remaining mental illness types were 12% to 16%.

All mental illness types had a higher prevalence of ever smoking tobacco daily over the follow-up than the reference cohort of never having a mental illness (ORs ranging from 2.0-6.5) (Table 2). The strongest association was evident for those diagnosed with bipolar disorder (OR = 6.5; 95% CI, 3.1–13.4), followed by those diagnosed with schizophrenia (OR = 4.7, 95% CI: 2.3–9.9) and those diagnosed with depression (OR = 3.0, 95%: 2.5–3.7).

For the remaining metabolic risk factors of high blood pressure and high cholesterol, there were no statistically significant differences between those with or without mental illness, including by type of mental illness (see Table 2). The reference cohort had a prevalence of high blood pressure of 17%, whereas the remaining mental illness types had a prevalence range of 10% to 19%. Similarly, the reference cohort had a prevalence of ever having high cholesterol of 10%, whereas, for mental illness types, this figure ranged from 5% to 12%.

The proportion of patients with care plans was lowest among those with schizophrenia (33%) and ADHD (35%) and highest among those with anxiety (51%) and depression (49%) (Table 2). The distribution of care plan formulation was similar across age and sex. Patients living in areas with the lowest SES had the highest proportion of care plans (54%). However, there was no evident socio-economic gradient from high to low SES, with similar proportions of care plans evident for each SES group.

For any selected mental illness, care plans were associated with lower odds of obesity (OR = 0.17, 95% CI, 0.05–0.60) and tobacco smoking (OR = 0.7, 95% CI: 0.6–0.99). Stratifying by selected mental illness type resulted in small numbers and insufficient data for some groups. However, the strongest OR magnitude for obesity was for those diagnosed with anxiety, and there were suggestive decreases in average BMI score for those diagnosed with schizophrenia (Beta = −6.6, 95% CI: −17.7; −4.5) and also lower odds of tobacco smoking for those diagnosed with bipolar disorder (OR = 0.5, 95% CI: 0.1–3.1) and ADHD (OR = 0.3, 95% CI: 0.03–2.9) (Table 3).

**Table 3.** Assessing the association between care plans (CP) among those with an active mental illness and those without mental illness in reducing metabolic risk, adjusted for Age and social Disadvantage.

	Average no. of CPs for those under CP	GP visits under CP	Obese (OR)	Change in BMI (*β*)^a^	Daily tobacco smoker (OR)	High BP (OR)	High chol (OR)	Change in total chol (*β*)^a^
Any mental illness	1.1	31.8%	0.2 (0.1; 0.6)	–1.9 (−13.8; 10.0)	0.7 (0.6;0.99)	1.3 (0.8;1.9)	1.2 (0.6:2.7)	0.09 (−0.04; 0.22)
Anxiety	1.1	26.3%	0.2 (0.1; 0.7)	–1 (−2.4;0.4)	0.8 (0.6;1.1)	1.2 (0.7;2.1)	1.6 (0.6;4.1)	0.09 (−0.07;0.24)
Bipolar	1.3	23.2%	^b^	0.3 (−8.4;9.1)	0.5 (0.1;3.1)	0.4 (0;12.2)	All on CP Have HighChol	0.33 (−0.72;1.38)
Depression	1.2	23.6%	1 (0.1; 6.7)	–0.5 (−2.6;1.6)	0.8 (0.5;1.3)	1.6 (0.8;3.3)	0.6 (0.2;2.5)	0.11 (−0.11;0.32)
Schizophrenia	1.2	21.4%	^b^	–6.6 (−17.7;4.5)	1 (0.1;9.8)	1.2 (0;48.8)	All on CP Have HighChol	–0.3 (−1.42;0.83)
ADHD	1.5	23.5%	^b^	2.1 (−9.1;13.2)	0.3 (0.03;2.9)	All on CP have High BP.	^b^	0.23 (−1.05;1.51)

Data from 2016 till 2023. ^a^ Beta-coefficients for Random Effects Linear Regression model. ^b^Insufficient variation in data. ORs presented from Random Effects Logistic Regression models compare those with a care plan to those without one.

**Figure 1.**
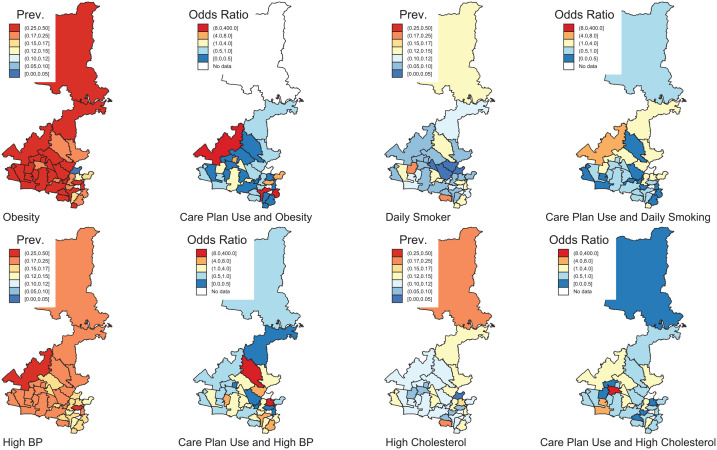
Prevalence of metabolic risk and association with care plan effectiveness (<1 OR) in modifying metabolic risk-adjusted for age and social disadvantage. Prev.: Period Prevalence over the follow-up of the metabolic risk factor among patients with active mental illness in that area of Western Sydney. Odds Ratio: Odds Ratio of care plans reducing the risk factor in patients with mental illness.

The prevalence of obesity and daily tobacco smoking was also higher among patients with mental illness residing in lower SES areas than the highest SES area (ORs~2.0). However, no SES differences were evident for the prevalence of high cholesterol and high blood pressure (Table 4). Area-based differences in the prevalence of metabolic outcomes and the impacts of care plans suggest that care plans were associated with reductions in obesity among patients with all selected mental illness types for all areas, except the western area of the Western Sydney PHN geographic catchment and also small areas in the south-east (Figure 1). Similarly, the odds of daily tobacco smoking due to care plans have also declined in the southern part of Western Sydney and the northern part of Western Sydney. The North and the West sides of Western Sydney have benefited from care plans in reducing high blood pressure. Similarly, all areas except the part of the west of Western Sydney and a few other pockets have seen a reduction in the odds of high cholesterol.

**Table 4.** Association of social disadvantage and metabolic factors in patients with mental illness from 2016 till 2023.

Metabolic risk factor	Obese		Daily tobacco smoker		High cholesterol		High BP	
SEIFA quintile (ref: 5 most advantaged quintile)	Prev.	OR	Prev.	OR	Prev.	OR	Prev.	OR
Low SES	30%	2.2 (1.8–2.7)	10%	2.0 (1.9–2.1)	11%	0.8 (0.6–1)	17%	0.98 (0.92–1.05)
2	31%	2.4 (1.9–3.1)	10%	2.1 (2–2.2)	11%	0.9 (0.7–1.1)	17%	0.99 (0.92–1.07)
3	25%	1.1 (0.9–1.3)	11%	2.2 (2.1–2.3)	10%	0.7 (0.6–0.9)	18%	1.10 (1.03–1.18)
4	25%	1.1 (0.9–1.4)	8%	1.5 (1.4–1.6)	11%	0.8 (0.7–1)	18%	1.12 (1.05–1.2)
High SES	24%	1.0	6%	1.0	12%	1.0	17%	1.0

Prev.: Period prevalence over the follow-up of the metabolic risk factor among patients with active mental illness in Western Sydney.

## Discussion

This study found higher obesity among those with schizophrenia and bipolar disorder compared to those presenting to primary health care without a mental illness. Daily tobacco smoking was also higher among those with all the types of mental illness investigated: schizophrenia, bipolar disorder, ADHD, anxiety and depression. There were negligible differences between those with a mental illness (compared to those without a mental illness) for high cholesterol and high blood pressure. These findings may suggest more effective management of metabolic outcomes amenable to widely accessible pharmaceuticals but less effective management of outcomes associated with behavioural approaches to prevention and control (e.g. obesity and tobacco smoking).

Furthermore, this study showed a social gradient in the prevalence of obesity and tobacco smoking among patients with mental illness, but not for tobacco smoking and blood pressure. This finding adds to the complexity of alleviating obesity and tobacco smoking through public health prevention and clinical management among lower socio-economic groups.

A key finding was that care plans were associated with lower odds of obesity and tobacco smoking among those with any mental illness. Analyses by selected mental illness types suggested that care plans were likely to reduce obesity in patients with schizophrenia, who are themselves at the highest risk of obesity among patients with mental illness: the magnitude of average increased risk of obesity among those with schizophrenia was similar to the magnitude of the average reduced risk associated with a care plan among those with schizophrenia. Furthermore, even though patients with any of the investigated mental illnesses had increased odds of tobacco smoking, care plans reduced the odds of tobacco for the cohort with mental illness as a whole.

Care plans were also associated with a decreased risk of metabolic outcomes for specific geographic areas within the PHN, notably higher socio-economic status areas in the northern and south-eastern parts of the PHN. This is consistent with studies showing a socio-economic gradient for metabolic outcomes, where socially advantaged people (and areas) have a reduced metabolic risk, and the converse is also true (Blanquet et al., 2019; Sommer et al., 2015).

The findings from small geographic regions in the PHN can help inform improved care planning for specific areas of Western Sydney and optimise the potential benefits of care plan interventions. The geographic pattern of the prevalence of metabolic risk outcomes and potential effectiveness (or otherwise) of care plans likely reflects the socio-demographic characteristics of these Western Sydney suburbs regarding socio-economic status and socio-cultural factors, including ethnicity. Western Sydney has a diverse, multicultural population, with a high proportion of residents from non-English speaking or culturally and linguistically diverse backgrounds (NSW Government, 2016). In addition, behavioural factors associated with metabolic risk outcomes may be entrenched in some communities. At the same time, decreases in social disadvantage may ameliorate metabolic risk outcomes due to the gentrification of other communities in Western Sydney.

From the literature, to control metabolic risk in patients with serious mental illness, it is recommended that actions occur at screening, diagnosis, treatment and disease control. Screening should be carried out at regular intervals at the point of care, and diagnosis should be made early with appropriate counselling. Treatment options include lifestyle change and medication that can be initiated and progressed using motivational interviewing techniques (Murphy and Daumit, 2023).

Preliminary evidence from a prospective cohort study in a sample of 83 patients with serious mental illness who received telephonic motivational interviewing indicated a 17% reduction in tobacco smoking rates and triglyceride level reduction from 2.2 to 1.8mmol/L (high to near normal levels) (Ringen et al., 2018). Physical activity could not be improved in those with negative symptoms, but could be improved in those with well-managed mental illness. Treatment should attempt to achieve evidence-based goals over the full range of metabolic risk factors. Tertiary options for disease control should be made available and accessible for any patient without serious mental illness (Murphy and Daumit, 2023).

Care plans have a major role in this therapeutic process of reducing metabolic risk from screening to disease control (Murphy and Daumit, 2023; Victorian Department of Health, 2023). Using care plans, GPs can act as an interface between a complex health system and a wary patient who may feel neglected or misunderstood. This is especially true for a patient population with potentially limited insight into mental health and ensuing metabolic mental health outcomes. The government significantly funds care plans (Department of Health and Aged Care, 2024) and present an opportunity to improve care for socially disadvantaged patients with serious mental illness. However, this study indicates that while care plans show promise, they must be utilised more frequently to show potential reductions in daily tobacco smoking rates. This study recognised the potential for care plans in facilitating tobacco smoking reduction. However, from this study, patients with mental illness have an absolute increase of 5% from 9% to 14% in daily tobacco smoking rates compared to patients without mental illness. Furthermore, in this study, on average, one in four patients with schizophrenia and bipolar disorder smoke daily. Focus should continue on patients with psychotic disorders (schizophrenia and bipolar disorder) and obesity, mainly due to the medication they take and the relatively increased social disadvantage they face due to the illness and otherwise.

Specific factors that may act on the effectiveness of care plans in achieving optimal metabolic outcomes include the social disadvantage of the patient, fluency of English, family and social supports and culturally and linguistically diverse background of the patients. (Burt et al., 2014) Other risk factors that affect metabolic risk include existing dietary and physical activity levels, education backgrounds, career status, health literacy and insight into their mental illness, personal finances to pay for treatment, access to services that are nearby or accessible via public transport and, importantly, continuity of care and compliance with the GP and mental health clinicians (Burt et al., 2014). All these factors are downstream of social disadvantage, and hence, management of patients with mental illness to prevent metabolic outcomes requires tailored care planning for social disadvantage. These could include more free sessions, higher remuneration to the GP or mental health clinician for providing free care to patients, vouchers for exercise and sport, and potentially discounted access to fresh food from social organisations that provide fresh food, which has been rejected from supermarkets because of appearance.

A recent review found that there is a lack of delineation of care regarding metabolic risk between primary care and mental health care (Murphy and Daumit, 2023). Time for screening of metabolic risk is needed for the patient with serious mental illness, and this can be difficult for both GP and mental health carers. Care plans represent a potential solution, requiring regularly updated documentation alongside regular health check initiatives (Zwar et al., 2007). The time taken for a care plan can allow the GP to overcome communication difficulties and assess the capacity of patients with severe mental illness (Department of Health and Aged Care, 2024). However, the drivers for the uptake and sustained engagement with primary health services need to be considered, as indicated by the differential associations across geographic areas in this study.

Strengths of this study include the demographically diverse population of Western Sydney, which has a high population burden of mental illness. There is a diverse range of social disadvantages and social advantages to make broad inferences on socio-economic differences in mental illness and metabolic outcomes. Data from WSPHN are maintained regularly for reporting purposes to multiple stakeholders, including the Federal Department of Health. The care plan is recorded accurately as an MBS item number, care plans are concrete in their requirements, and compliance is required to obtain funding from the federal government. This is likely to minimise misclassification bias in both the exposure and outcome. The study is also limited to data on patients who attend a GP. However, in Australia, GPs are the primary initial referral point in the health system, with 78% of individuals attending a GP at least once per 2 years in Western Sydney, which has a population of 1.1 million. This unpublished calculation is based on internal medical records and the Australian Bureau of Statistics census data. GPs are often the first clinical point of contact for patients with mental illness. A GP visit is also required for referral to specialist care and most primary mental health care services.

The study’s limitations relate to the irregular updating of data on metabolic factors when a patient visits a GP even if there is an opportunity to do so and based on screening guidelines. Irregular updates of metabolic measures were observed throughout the PAT-BI dataset used in this study. GPs are requested to collect information on the frequency of screening for metabolic risk through government guidelines (Victorian Department of Health, n.d.). Furthermore, this study only reports on available and regularly recorded metabolic indicators in the PAT-BI electronic medical record system, so measures such as waist circumference could not be reported on. It is difficult to measure the level of engagement a patient has with their GP and the associated care plan without more in-depth qualitative approaches to understand patient experiences and drivers of engagement with services. Confounders were also limited to variables routinely collected in the dataset, including age, gender, and area socio-economic status. The measurement of social disadvantage was at the level of the area of the GP clinic rather than the residence of the patient, but this will likely reflect the area SES of surrounding neighbourhoods. Other unmeasured confounders may include cultural and linguistic diversity, Indigenous status and access to family or social supports. Also, a few associations have wide confidence intervals since they were based on small numbers of cases.

Patients with mental illness are at increased metabolic risk due to social disadvantage, their circumstances and their journey on the recovery path, including treatment itself. This risk increased dramatically as social disadvantage, education and lack of fluency in English increased. Care plans to reduce the metabolic risk for patients with serious mental illness should be tailored for the circumstances of the patient to reflect social disadvantage, lack of social support and cultural diversity, which all affect participation in the community and the maintenance of a healthy diet and optimal physical activity. Psychotic disorders, due to the treatment and negative symptoms, require extended and focused care planning in a multi-disciplinary team setting due to increased metabolic risk (including from treatment) and the challenging nature of their circumstances, mental illness and compounded social disadvantage.

## Supplemental Material

sj-docx-1-anp-10.1177_00048674251337030 – Supplemental material for Improving metabolic risk in patients with mental illness through ‘mental health care plans’ in primary health care Supplemental material, sj-docx-1-anp-10.1177_00048674251337030 for Improving metabolic risk in patients with mental illness through ‘mental health care plans’ in primary health care by Mithilesh Dronavalli, Andrew Page, Shahana Ferdousi, Max Osaghae and Sandro Sperandei in Australian & New Zealand Journal of Psychiatry
