# Supplementary material for: Improving metabolic risk in patients with mental illness through ‘mental health care plans’ in primary health care
Source: Aust N Z J Psychiatry. 2025 May 13;59(8):692–701. doi: 10.1177/00048674251337030 (PMC12280240; doi:10.1177/00048674251337030)
Supplement: sj-docx-1-anp-10.1177_00048674251337030 – Supplemental material for Improving metabolic risk in patients with mental illness through ‘mental health care plans’ in primary health care [file sj-docx-1-anp-10.1177_00048674251337030.docx]

Supplementary Table 1

| Mental Illness | Active or Inactive | Inactive at One Stage | Active at one stage | Both Active and Inactive at different stages |
| --- | --- | --- | --- | --- |
| Anxiety | 68227 | 50066 | 19894 | 1733 |
| Depression | 42235 | 10049 | 32892 | 706 |
| Bipolar | 3679 | 3208 | 567 | 96 |
| Schizophrenia | 4103 | 3735 | 473 | 105 |
| ADHD | 3794 | 3186 | 668 | 60 |

Supplementary Table 2

|  |  | Never had Mental Illness |  | Active Mental Illness |  |
| --- | --- | --- | --- | --- | --- |
| Data Subset | Variable | Total Cohort (n) | Percent Missing (%) | Total Cohort (n) | Percent Missing (%) |
| Obesity | Obese | 275,585 | 71.56 | 3,000 | 70.33 |
|  | Care Plan | 275,585 | 0 | 3,000 | 0 |
|  | Age | 275,585 | 1.43 | 3,000 | 0.03 |
|  | SEIFA | 275,585 | 31.77 | 3,000 | 4.2 |
|  | Sex | 275,585 | 1.06 | 3,000 | 1.3 |
| Daily Tobacco Smoking | Daily Tobacco Smoker | 342,237 | 0 | 4,903 | 0 |
|  | Care Plan | 342,237 | 0 | 4,903 | 0 |
|  | Age | 342,237 | 1.23 | 4,903 | 0.02 |
|  | SEIFA | 342,237 | 30.47 | 4,903 | 3.79 |
|  | Sex | 342,237 | 0.91 | 4,903 | 0.96 |
| High Cholesterol | HighChol | 607834 | 0 | 1,279 | 0 |
|  | Care Plan | 607,834 | 0 | 1,279 | 0 |
|  | Age | 607,834 | 0 | 1,279 | 0 |
|  | SEIFA | 607,834 | 96.56 | 1,279 | 0.86 |
|  | Sex | 607,834 | 0.11 | 1,279 | 0.23 |
| High BP | HighBP140 | 333,324 | 47.64 | 4,577 | 49.29 |
|  | Care Plan | 333,324 | 0 | 4,577 | 0 |
|  | Age | 333,324 | 1.19 | 4,577 | 0.02 |
|  | SEIFA | 333,324 | 31.42 | 4,577 | 4.26 |
|  | Sex | 333,324 | 0.93 | 4,577 | 1.07 |

Supplementary Table 2 indicates missing data for the analysis of each outcome.

Supplementary Table 3

|  | Present SEIFA | Missing SEIFA |
| --- | --- | --- |
| **Outcomes** |  |  |
| HighChol | 9.4% | 10.4% |
| HighBP | 16.7% | 17.5% |
| Obesity | 34.1% | 32.9% |
| DailySmoker | 10.7% | 10.1% |
| **Exposure** |  |  |
| Active or Inactive Mental Illness | 4.5% | 0.1% |
| Active Anxiety | 2.3% | 0.1% |
| Active Bipolar Disorder | 0.1% | 0.0% |
| Active Depression | 1.2% | 0.0% |
| Active Schizophrenia | 0.1% | 0.0% |
| Active ADHD | 0.1% | 0.0% |
| Confounders |  |  |
| Age | 45.40 | 43.90 |
| Sex |  |  |
| 0 | 56.6% | 55.6% |
| 1 | 43.4% | 44.4% |
| 2 | 0.0% | 0.0% |

Supplementary Table 3 showed that while Postcode data and hence SEIFA values are sparse the data is likely to be missing completely at random as outcome/exposure/confounding variable values are not differentially biased by presence or missingness of SEIFA.
